# Book Review

**Published:** 1898-12

**Authors:** 


**Elements of Latin.** By GEO. D. CROTHERS, M.D., and HIRAM H. BICE. Pp. xii., 242. Philadelphia: The F. A. Davis Company. 1898.—The Yankees are nothing if not original. Poor old Balbus, the friend of our early Latin struggles, is here deposed: perhaps he emigrated to the States, and suffering from appendicitis, fell a victim after the universal operation. Students of medicine and pharmacy who neglected the study of the dead languages in their school days are to remedy their deficiencies in a new way. The unfortunate student is now to be taught by sentences that will impress on him some medical, anatomical, or therapeutic truth. But the method of treatment formulated in "*Femina aegrota misturam asafoetidae habet*" is, perhaps, a little too wide for general application. He is also gravely told that "*Tabacum ab multis Americanis masticatur; gummi ab multis puellis.*" We always believed that the charge of chewing tobacco was indignantly denied, and should not "*Americanis*" be "*civibus Civitatum Foederatarum*" or has the Monroe doctrine extended to Canada, which we believe still remains part of the British Empire? Then we have the useful sentence "*Pelvis feminae est lata,*" at once conveying an anatomical truth with "very choice Italian." The list of anatomical proper names is interesting, though many are included that can hardly be said to be in common use. There are a few errors. The christian name of Glisson was Francis, not Francois. It seems curious that the birth and death date as well as the Christian name of Cohnheim are not given; they were 1839—1884, and Julius.

**State Aided *v.* Voluntary Hospitals.** By W. Knowsley Sibley, M.D. Pp. 16. London: T. Burleigh. 1896.—We recommend this little pamphlet to all who are interested in the question of hospital management. Dr. Sibley shows how lamentably weak our system of hospital relief is when compared with that of other nations, and though the information given of the working details is very meagre, many practical lessons are to be learnt from the German, French, and Swedish methods. The latter part is devoted to an exposition of the abuses existing in English hospitals, of which we have heard so much of late.

**Some Incidents in General Practice.** By Augustin Prichard. Pp. 93. Bristol: J. W. Arrowsmith. 1898.—Not very long before his death Mr. Augustin Prichard published an interesting and instructive little book which we favourably reviewed at the time. It gave a graphic description of Mr. Prichard's early life and education for the medical, or rather surgical, profession. In the present book there is a plain and straightforward account of many of the ups and downs of medical life, mainly those which occur in the busy routine of the general practitioner. Mr. Prichard's description of his earlier experiences in general practice is given in a charmingly unaffected manner. His favourite speciality in surgery was ophthalmology, and this he worked at very diligently quite from the beginning of his professional life, chiefly amongst the poor who came in numbers to the Eye Dispensary of his uncle, Mr. Estlin, in Frogmore Street. Mr. Prichard seems to have been very much amused with their eccentricities, and with the queer liberties the patients took with their native tongue, either spoken or written; and at the beginning of his practice, "noticeable particularly at the Eye Dispensary and Infirmary, was the number of persons who could neither read nor write; and when asked to spell their names, invariably made the same reply, 'I ain't no scholar.'" The improvement in dress and personal cleanliness amongst the poor as represented in the out-patient departments of public charities is remarkable. "You now seldom or never see a man in absolute rags and tatters not enough to cover him, and with bare feet, unless he assumes it for his own purposes." Dr. James Cowles Prichard, the celebrated physician, the father of Mr. Augustin Prichard, was a great authority on insanity and published an excellent treatise on mental diseases. In consequence of this he was, towards the end of his life when retiring from private practice, appointed a Government Inspector of Lunatic Asylums, and most ably did he perform his important duties. Mr. Prichard, no doubt, often assisted his father in this work, and he gives a graphic account of his long tiresome journeys and of some amusing interviews and occasionally awkward predicaments in which he was placed by the lunatic patients. Mr. Prichard has described all this *con amore*, and it is, perhaps, the most interesting part of the book and should on no account be missed by the reader. A portrait of Dr. J. C. Prichard forms the frontispiece to the book, which also contains, from a sketch by Mr. Augustin Prichard, a good picture of the Bristol Cathedral before the present alterations and improvements. It was quite worth Dr. James E. Prichard's while to issue this record of his father's experiences.

**Exercises in Practical Physiology.** Part I.—*Elementary Physiological Chemistry*. Pp. 24. Part III.—*Physiology of the Nervous System. Electro-Physiology*. Pp. 91. By AUGUSTUS D. WALLER, M.D. London: Longmans, Green, and Co. 1897.—These little books are meant primarily for the use of students at St. Mary's Hospital. The first part is not much more than a catalogue of experiments to be done in physiological chemistry, and contains a minimum of explanation. The third part bears special testimony to Dr. Waller's work in electro-physiology.

**Lexique-Formulaire des Nouveautés médicales et biologiques.** Par PAUL LEFERT. Pp. 336. Paris: J.-B. Baillière et Fils. 1898.—Information which is scattered through a large number of treatises and journals, but which is not yet incorporated with the text of even the newest text-books will be found in this volume. It contains an outline of the newest work in all the departments of scientific medicine and surgery, arranged in dictionary form, with the names of authors, the names of diseases, and the names of medicines so arranged by Professor Paul Lefert, that the volume will be of service on the table of the practitioner as well as in the library of the student. We notice an excellent description of the agglutination of the microbes which forms the Widal test for typhoid.

**The Medical Examination for Life Assurance.** By F. DE HAVILLAND HALL, M.D. Pp. 73. Bristol: John Wright & Co. 1898.—An article on the selection of lives for life assurance published in the *Medical Annual* for 1896, met with so favourable a reception that this small book was devised as likely to be useful to those practitioners who do not consult larger works on the subject. The author did well to make the book a small one; there is no need for lengthy treatises on this topic, and yet some training is required. The problem whether a particular person is healthy is always one which any medical man of mature judgment can solve; but the classification of defective lives needs consideration, and opens up many difficult problems. We think this book is an excellent guide for those whose views on the question are not matured, and the author does not make mysteries out of trifles.

**Differential Diagnosis and Treatment of Coma.** Arranged by George A. Huntley, M.D. Weston-super-Mare: Huntley Bros. [n.d.].—This chart classifies the characteristics of no less than thirteen varieties of coma, indicates the appropriate treatment for each, and adds that a fourteenth variety, feigned coma, often misleads the most practical expert. It is correct as far as it goes, and if hung up for reference would be found more instructive than an ordinary wallpaper.

**Yellow Fever in the West Indies.** By Izett Anderson, M.D. Pp. xv., 106. London: H. K. Lewis. 1898.—After retirement from active practice Dr. Anderson looked over his old notes, and thought that a record of his experience of over thirty years might benefit practitioners in the West Indies. His book is entirely clinical in its scope, and does not attempt to deal with the pathology and bacteriology of the disease. He says "I have never met with a single case in which I thought yellow fever had been contracted by either mediate or immediate contact with a previous case, or with a patient's exhalations or excreta;" yet, nevertheless, he thinks "it will probably conduce to the mental tranquillity, and enhance the reputation of the practitioner, if he treats all his cases of this disease, as if they were of the most contagious character." Dr. Anderson's dedication of his little book to his "dear brothers" leaves it uncertain whether he means to honour some members of his own family or his professional brethren.

**Aneurysms of the Aorta.** By Oswald A. Browne, M.D. Pp. 38. London: H. K. Lewis. 1897.—This is a careful and laborious examination of 173 records of necropsies on cases in which aneurysm of the aorta was present. The cases are tabulated, and consequently the leading features can be readily seen by anyone wishing to gather evidence upon any special point. On glancing down the list which includes aneurysms of the ascending arch of the aorta, we notice that of eighteen cases of death from rupture no less than nine ruptured into the pericardial cavity. This is largely due to the great frequency of aneurysms situated immediately above the aortic valves. The work, which was presented as a thesis for the Cambridge M.D., does not include examination of clinical records; but it would have been interesting to have known how many of these aneurysms were suspected during life. It unfortunately often happens that the fatal rupture into the pericardium is the first indication of their presence. The tables illustrate forcibly the danger of asphyxia in cases of aneurysm of the transverse arch. Thus in twenty-one cases death is attributed to dyspnœa or asphyxia, and in only sixteen to rupture; whereas in aneurysm of the descending part of the arch, rupture may be said to be the only cause of death. Of eighteen cases in which the cause of death is mentioned, in sixteen there was rupture.

**Surgical Technics in Hospital Practice.** By K. W. MONSARRAT, M.B. Pp. 132. Bristol: John Wright & Co. 1898.—This small manual is "intended for junior men only" to systematise the routine duties of surgical hospital practice. Many of the remarks and instructions are good, particularly those referring to present-day asepsis, and the appendix of surgical rules for nurses. The merit, however, is unequal, for whilst the details of an important operation like tracheotomy are deliberately omitted, details of treatment in Kocher's excision of the tongue are given. In our experience, a nutrient enema of 5½ ounces with addition of meat peptone (p. 57) exceeds the normal rectal appetite. The book is not exhaustive, but may be read with profit in conjunction with text-books; details of the rarer operations, which vary with the operator, need not have been given.

**Notes of Thirty-two Consecutive Abdominal Sections, with Thirty Recoveries, Performed within the last 17 Months: with Observations.** By JAMES MACPHERSON LAWRIE, M.D. Pp. 66. London: John Bale, Sons & Danielsson, Limited. 1897.—This is the booklet form of a paper read at the Montreal meeting of the British Medical Association. A large bulk of the cases is composed of oöphorectomy and ovariotomy. The deaths occurred in cases of salpingitis and fibroid uterus. The term "cured" is too loosely applied to the cases of cancer of the uterus, as an insufficient time has elapsed in most of them to be sure that recurrence will not take place.

**The Tallerman Treatment by Superheated Dry Air.** Edited by ARTHUR SHADWELL, M.B. Pp. xi., 173. London: Baillière, Tindall and Cox. 1898.—Considering the price at which this book is sold, it is fairly obvious that it is produced at a loss, *i.e.*, that it is an advertisement of the apparatus. This being so, it is a little odd that it should be edited by a medical man. The next striking point is that the description of the apparatus is most meagre, and there is no illustration of it. It is further stated that the secret of the apparatus lies in "an ingenious arrangement for keeping the air really dry." Though no one can object to the apparatus being patented by the inventor and hired out at a profit, yet the methods of pushing it into notice hardly commend themselves. The method of treatment is, as shown by the results, most efficacious in many chronic joint diseases, such as gout, rheumatism, rheumatoid arthritis, and sprains.

**The Treatment of Sarcoma and Carcinoma by Injections of Mixed Toxins.** By C. MANSELL MOULLIN, M.D. Pp. 66. London: John Bale, Sons & Danielsson, Ltd. 1898.—Our own limited experience with the mixed toxins has not been attended with very satisfactory results; but we are pleased to see that the list of cases which the author has collected from various sources shows that there may still be a ray of hope for those afflicted with inoperable sarcoma. The author has wisely made a collection of cases of erysipelas occurring in the course of malignant affections and followed by disappearance of the growth. These show the *rationale* of the treatment in a convincing manner.

**Hunyadi János.** By Dr. E. MONIN. Pp. 113. Budapest: Andreas Saxlehner. 1898.—These essays on clinical hydrology are founded on the dictum of Stahl: "Plethora omnium morborum mater." They comprise the results of individual clinical investigation of the action of Hunyadi János water by Dr. Monin, of Paris, together with reprinted papers by Prof. Semmola and Dr. Sirotkine. The general conclusion is, that the Hunyadi János water is one of the best of saline purgatives, and has a great range of utility in the treatment of many diseases.

**Die Anatomie und Behandlung der Geburtsstörungen nach Antefixirung des Uterus.** Von Dr. W. RÜHL. Pp. 82. Berlin: S. Karger. 1897.—The author gives a minutely detailed account of the results as regards parturition of various methods of fixing the uterus in a position of anteversion, with the operative measures that may be necessary in order to effect delivery. In dealing with the anatomy of the pregnant uterus he brings out very clearly the facts which have important bearing on the subject of antefixation generally. The chief deductions which he draws from these are, that in performing vaginal fixation the sutures should be placed as low as possible, so that the expansion of the anterior uterine wall may not be interfered with, while ventro-fixation should be performed in such a way as not to produce firm adhesions. The most common disturbances in labour following fixation in cases where these cautions have been neglected are caused partly by the displacement of the long axis of the uterus, the angle which it makes with the plane of the brim being very much diminished, and the cervix lying near the promontory; this prevents the descent of the presenting part and causes a tendency to transverse positions, delivery being effected with difficulty either by forceps or version, the latter proceeding being very risky on account of the thinning of the posterior wall. To deal with more difficult cases an operation has been devised which consists in dividing the anterior lip of the cervix, part of the anterior vaginal wall, and the lower uterine segment after opening the anterior cellular interval between the uterus and bladder. The result of this is to make an opening into the uterus more nearly corresponding to the axis of the inlet and allow of the descent of the presenting part. After delivery the incision is sewn up. Abortions and miscarriages are shown to be frequent; but it is not absolutely demonstrated that these are due to the operations. Alexander's operation appears to have no ill-effects either on pregnancy or parturition. Dr. Rühl illustrates his points by several very clear diagrams.

**Polyneuritis in relation to Gestation and the Puerperium.** By H. G. TURNEY, M.D. Pp. 47. London: J. & A. Churchill. 1898.—The subject dealt with in this pamphlet, reprinted from *St. Thomas's Hospital Reports*, is one of great interest. The disease is rare; but from what we have seen of it we are inclined to agree with the German observers that it is usually of a septic origin, and is more common in patients who are predisposed to nervous affections.

**A Practical Textbook of the Diseases of Women.** By ARTHUR H. N. LEWERS, M.D. Fifth Edition. Pp. xviii., 526. London: H. K. Lewis. 1897.—This is a thoroughly practical book, and has been brought well up to date, though many details are necessarily omitted in such a work. The outlines of the subject are given in clear and concise language, supplemented by numerous illustrations. Tait's flap-splitting operation for ruptured perinæum is made more intelligible than in most gynæcological works; and we are pleased to notice an absence of the anatomical minutiæ with which similar treatises are frequently padded.

**Outlines of the Diseases of Women.** By JOHN PHILLIPS, M.D. Second Edition. Pp. xvi., 275. London: Charles Griffin & Company, Limited. 1897.—The first edition of this book was published about four years previously, and there is little difference in the two editions beyond the inclusion in the present volume of short notices on kraurosis, deciduoma malignum, movable kidney, and a few other subjects. The writing as a whole is compressed into as small a space as is compatible with clearness. The book, of course, lacks the detail found in larger manuals, to which it forms an useful adjunct. A curious error occurs in the middle of page 55, where a whole line has been duplicated.

**Das Studium der Frauenheilkunde ihre Begrenzung innerhalb der allgemeinen Medicin.** Von A. MACKENRODT. Pp. 35. Berlin: S. Karger. 1898.—This is the first of a series of studies in gynæcology and obstetrics which are being issued by Dr. Mackenrodt and his assistants in his private hospital for women. As an introductory lecture on the need and importance of a special training in the diseases of women, this plea from a writer full of enthusiasm for his life's work will be found worthy of a careful reading.

**Über die Resultate der Radical-behandlung des Gebärmutter-Scheidenkrebses mit dem Glüheisen.** Von Dr. GEORG GELLHORN. Pp. 92. Berlin: S. Karger. 1898.—This monograph treats of the extirpation of the uterus for cancer by means of the electric and actual cauteries. The instruments and methods are described, and it is claimed that the results obtained compare favourably with other methods.

**Sur trois Cas de Complications intra-craniennes d'Origine otique.** Par le Dr. E. J. Moure. Pp. 16. Bordeaux: G. Gounouilhou. 1897.—**Des Adénoïdites chez les Adultes.** Par le Dr. E. J. Moure. Pp. 8. Bordeaux: Feret et Fils. 1898.—**Sur le Traitement des Sinusites (maxillaire excepté).** Par le Dr. E. J. Moure. Pp. 32. Bordeaux: Feret et Fils. 1898.—Dr. Moure, of Bordeaux, is a prolific writer on matters relating to diseases of the larynx, ear, and nose, but he always writes with knowledge and we welcome the present additions to the list of his pamphlets. Surgeons in France have hitherto been somewhat behind those of England and America in operating for intra-cranial complications of ear-disease, but Dr. Moure now shows that they are becoming fully alive to the importance of early operation in these cases. Dr. Moure maintains that adenoids are more commonly met with in adults than is generally supposed, and he gives some interesting cases which seem to prove his point. The difficult subject of sinusitis in the ethmoidal, frontal, and sphenoidal regions is clearly dealt with and the treatment fully described.

**Outlines of Rural Hygiene.** By Harvey B. Bashore, M.D. Pp. v., 84. Philadelphia: The F. A. Davis Company. 1897.—Dr. Bashore has utilised his experience as Inspector for the State Board of Health of Pennsylvania in writing a short handbook intended to correct the almost absolute neglect of sanitary rules in districts outside of the great cities. His recommendations seem generally sound, and some of the illustrations are original and suggestive.

**Reports from the Laboratory of the Royal College of Physicians, Edinburgh.** Vol. VI. Edinburgh: William F. Clay. 1897.—One of the most important papers from a practical point of view in this volume is one by Dr. Dunlop on the excretion of oxalates in the urine. He shows that it is invariably due to oxalates in the food, and that oxalates are not produced within the animal body. Thus there are no oxalates excreted on a diet of milk and meat, as it is the vegetables which are their source. The condition of oxaluria is essentially one of hyperacid dyspepsia, in which more oxalates than usual are dissolved during digestion and pass into the blood; and the condition can be cured by the administration of either acids before meals or alkalies after. This research thus clears up a question which is interesting theoretically and has most important practical bearings. Besides this paper there are, as might be expected, many others of great interest. Dr. Berry Hart shows that the vagina is formed from the Wolffian ducts, and not, as hitherto thought, from the Müllerian. The various cases of atresia and abnormalities of the lower part of the genital tract thus receive a more satisfactory solution than was before possible. There are some valuable papers by Dr. Stockman on the amounts of iron in the tissues in various kinds of anæmia. The volume takes very little more time to read than a number of many a weekly medical journal; but whereas in the former one finds in every paper new and suggestive work, in the latter, as a rule, the amount of fresh knowledge is infinitesimal. And yet everyone reads a weekly journal.

**The Johns Hopkins Hospital Reports.** Vol. VI. Baltimore: The Johns Hopkins Press. 1897.—This volume contains a report in neurology by Dr. Henry J. Berkley, which consists of a study of the lesions produced by the action of certain poisons on the cortical nerve cell. The poisons dealt with are acute and chronic alcoholic poisoning in rabbits, serum poisoning, acute and chronic ricin poisoning, and the toxin of experimental rabies. The stains used in these investigations were Nissl's and the silver phospho-molybdate, a modification of Golgi's method devised by the author. It is hardly necessary to mention the great advance that these methods of staining have already made in the knowledge of the pathology of the nerve cell, and it is impossible to deal here in detail with Dr. Berkley's researches. It must suffice to say that the paper forms a very valuable and important contribution to nervous pathology, which should be carefully read and studied. We cannot, however, refrain from calling attention to the excellence of the illustrations, which are quite remarkable. They show that the preparations must have been very beautiful ones, and the way in which the reproductions have been done deserves the highest praise. Three papers dealing with uterine affections follow, and then there is an elaborate investigation by Dr. Wm. D. Booker, entitled "A Bacteriological and Anatomical Study of the Summer Diarrhœas of Infants." This paper has involved an enormous amount of labour, 92 cases of various forms of summer diarrhœa in infants having been carefully studied. Of 33 cases in which the disease terminated fatally, a full account is given of the pathological changes found in the organs and of the results of a thorough bacteriological investigation in each case. The results attained in all the cases are summarised at the end of the paper. From the correspondence between clinical symptoms, bacteriological results, and anatomical changes existing in many cases, the author distinguishes three principal forms of summer diarrhœa in infants: (1) dyspeptic or non-inflammatory, (2) streptococcus gastro-enteritis, and (3) bacillary gastro-enteritis; but we must refer our readers to the paper itself, which will well repay perusal. There is a series of fine plates of the microscopical appearances. The concluding paper is again a very valuable one, entailing much painstaking research, and is by Dr. Simon Flexner. It deals with the pathological changes produced in the organs by toxalbumin intoxications. The poisons the action of which is investigated with this object are experimental diphtheria, streptococcus (these two acting in combination), cholera vibrio, and acute and chronic abrin and ricin intoxications. The paper is a very complete one, dealing with the literature of the subject, as well as a long series of original investigations; it also contains a section on the lesions in man produced by certain toxic substances, and a concluding one on the pathogenesis and significance of the lesions of intoxication. It is impossible here to give an adequate account of Dr. Flexner's researches, the paper itself must be read; like the preceding papers, it is well illustrated. Enough has been said to indicate the wealth of matter to be found in this volume and the high standard of the several papers which compose it.

**St. Thomas's Hospital Reports.** New Series. Vol. XXV. London: J. & A. Churchill. 1897.—This volume contains a number of interesting papers and reports in detail of the work of the various departments of the Hospital. These latter are very carefully done, and as there are short abstracts of the cases of most interest and importance they form a very valuable storehouse of clinical records and statistics. The mortality of 107 cases of diphtheria treated with antitoxin in 1896 is 36.44 per cent., and it is to be noted that of those treated on the first day of the disease the mortality is only 15.38 per cent. The mortality of the cases of enteric fever was 11.2 per cent. There is a special table of cases of pyæmia and septicæmia; and in the Gynæcological Report short outlines of abdominal operations are given, with three special tables of those undertaken for diseases of the ovaries, and of the Fallopian tubes, and for conditions other than these. There is a special report of the newly-established X-ray department. Dr. Turney's paper on "Polyneuritis in Relation to Gestation and the Puerperium" has been reprinted in pamphlet form, and is noticed separately on page 352. Dr. Acland has a well-reasoned and temperate article on compulsory vaccination; and amongst other papers of interest there is one on surgical bilharziosis as seen in Egypt, by Mr. H. Milton, who has had a large experience of the disease in that country; an analysis of 26 cases illustrating the clinical symptoms of tubal gestation in the early months, by Dr. Walter W. H. Tate; a very useful and practical paper by Mr. H. H. Clutton on 47 cases of cleft palate, of which 41 were successful and 3 partially so; two remarkable cases of abdominal actinomycosis, by Mr. Makins; a paper on hydronephrosis, by Mr. Battle; and other papers which will well repay perusal.

**Transactions of the British Institute of Preventive Medicine.** First Series. London: Macmillan & Company, Limited. 1897.—This volume consists of a collection of original investigations by members of the staff of the Institute, and is appropriately prefaced by a short account of the main objects of its foundation from the pen of Lord Lister. The transactions open with a short paper on the relations of streptococci from various sources; then follows a carefully worked out series of experiments apparently establishing the identity of the pseudo-diphtheria bacillus with the Klebs-Löffler organism. In view of the extreme importance of the matter, and of the disappointing results that have attended parallel investigations, notably in the case of anthrax and typhoid, it is necessary to await independent corroboration before accepting the authors' conclusions unreservedly; but the very careful and seemingly conclusive observations recorded are certainly worthy of the immediate attention of other specialists in diphtheria. The volume includes also an elaborate note on the culture and reactions of the micrococcus gonorrhœæ and its companions, an account of a simple and effective process for the sterilisation of milk, and a lengthy investigation of the value of a well-known filter in connection with the sterilisation of water. An account of the bacteriological examination in a case of bubonic plague, an inquiry into the bacterial flora of dust particles, and a note on the preservation of water organisms in water instead of the usual culture media, complete a volume which bears testimony to the valuable work carried out in the laboratories of the Institute in spite of the adverse influences which have crippled its powers. It is to be hoped that under new and more favourable conditions this work will be largely extended.

**Transactions of the Royal Academy of Medicine in Ireland.** Vol. XV. Dublin: Fannin and Co., Ltd. 1897.—Many papers of much interest will be found in this volume. The opening address upon the development of the brain, by Professor Wilhelm His, of Leipzig, is the work of a leader in anatomy, and it is exceedingly well illustrated. In the section of medicine, a paper by Dr. H. C. Drury shows that we have in guaiacol applied locally "another and a valuable weapon with which to attack" pyrexia. Numerous other papers on the various branches of medical science are well worthy of study, and show that the Royal Academy of Medicine in Ireland has ardent workers in many fields.

**Transactions of the Ohio State Medical Society.** Norwalk: The Laning Printing Company. 1897.—This volume marks a new departure, in that the papers and discussions on similar subjects are compiled into chapters. That on surgery includes papers bearing upon the application of aseptic surgery to general practice and in the treatment of retention of urine, which are worthy of careful reading. It includes also an interesting paper, by Dr. C. B. Parker, on the administration of pure oxygen in chloroform anæsthesia. We agree with the statement of the editor, Dr. Harvey Reed, that it is the duty of those concerned "to make each succeeding volume superior to its predecessor," and in this he has set a good example; but we would suggest that the head-lines should give the subjects of the pages.

**Transactions of the American Surgical Association.** Vol. XV. Philadelphia: William J. Dornan. 1897.—This annual volume is a storehouse of information for the operating surgeon. It contains the latest views of surgeons on the other side of the Atlantic on all matters relating to surgical diagnosis and treatment. The present volume is fully equal to its predecessors. It begins with the address of the president, Dr. J. Collins Warren, the subject of which is the "Influence of Anæsthesia on Surgery," and contains many valuable articles, among which we may mention "The Roentgen Rays in Surgery," by Dr. J. William White; "The Indications for and the Technique of Hysterectomy," by Dr. John Homans; and "Removal of the Gasserian Ganglion," by Dr. Stephen H. Weeks. The biographical articles include notices of the late Sir Spencer Wells, Sir John Eric Erichsen, and Sir George Murray Humphry, all of whom were honorary Fellows of the Association.

**Reports and Papers of the American Public Health Association.** Vol. XXII. Concord: Republican Press Association. 1897.—With such wealth of material—some fifty or sixty papers on various subjects—there is, as might be expected, much to interest and instruct in this volume. We have been especially struck by the "Statistics of Vaccination and of Mortality by Small Pox in the City of Mexico, from 1872 to 1895" given by Dr. José Ramirez, General Secretary of the Supreme Board of Health, who writes: "The Sanitary Code of the Republic only provides for revaccination in the army, and this provision is founded on experience, which shows that the vaccine has not degenerated in Mexico, and that one sole inoculation confers immunity from the disease. These curious and very important facts can only be explained by the other fact, that the vaccine has been preserved for a period of 88 years by five persons and that, having passed through so few hands, it has always been selected with unequalled expertness. . . . Revaccination has several times been attempted in the office of the Supreme Board, with the same negative results; but the fact that is best proved by this permanent immunity, is the extreme rarity of any case in which an adult succumbs to small pox. . . . Another significant fact is the great danger that foreigners who are vaccinated in their own country run of contracting fatal small pox in Mexico. These cases occur with great frequency, and the Board of Health has found itself obliged to invite foreigners to be revaccinated." There is no opposition to vaccination in Mexico, and the satisfactory results of efficient vaccination, which has been obligatory since 1891, are well shown in the appended tables. We trust this country may, in its own interests, long be spared the domination of "the conscientious objector."

**Transactions of the Epidemiological Society of London.** New Series. Vol. XVI. London: Shaw and Sons. 1897.—Following Professor Lane Notter's interesting address on "Infective Diseases in the Tropics," Mr. James Cantlie contributes an opportune and instructive paper on "The Spread of Plague." Dr. John C. McVail criticises with much force the dissentients' statement of the Vaccination Commission, and Dr. Niven discusses "The Prevention of Tuberculosis." Dr. Louis Parkes contributes "Observations on the Infectivity of Diphtheria," especially in relation to school-closure; Dr. Hamer writes on "Age-Incidence in Relation with Cycles of Disease-Prevalence," and Dr. Davidson discusses "The Seasonal Fluctuations of Epidemic Diseases." The volume closes with a memorandum about the Jenner Memorial Medal, and an obituary notice of the late Sir Benjamin Ward Richardson.

**Transactions of the Ophthalmological Society of the United Kingdom.** Vol. XVII. London: J. & A. Churchill. 1897.—Among other matters contained in this volume there are some very good cases of that peculiar condition, microphthalmos with cystic protrusion from the globe, reported by Mr. Treacher Collins, who has added a full account of the literature, and pathology as far as it has been determined, of the subject. Mr. Nettleship furnishes an exhaustive report on cases of central amblyopia as an early symptom of tumour of the chiasma. Based on researches from the bacteriological laboratory of Guy's Hospital, there is an excellent consideration and illustration of Tuberculosis of the conjunctiva, the classification of the various groups formulated by Sattler being adopted and followed out. Several very interesting cases of intraocular cysticercus with illustrations are also given. The discussion on retro-ocular neuritis, opened by Mr. Marcus Gunn and Dr. Buzzard, is very fully reported, and contains some very interesting facts and theories. This is a fine volume brimful of information to those who are interested in the study of ophthalmology.

**Transactions of the American Ophthalmological Society.** Vol. VIII., Part I. Hartford: Published by the Society. 1897.—Most of the cases in this volume are rare ones and well reported, while the illustrations, which are mainly taken from photographs, are all that could be desired. We think, however, that in reporting several of the cases too much space is given to the refraction where the refraction of the cases has no possible interest. Four cases of exophthalmos are quoted, due to different causes, and fairly well exhaust the subject. Diphtheritic conjunctivitis, according to Dr. Myles Standish, is only to be diagnosed by means of the bacteriological examination and not at all from the clinical appearances. From this view we dissent. Dr. Lucien Howe, in the treatment of an obstinate case, makes no mention of having tried quinine lotion, which, in this country at least, is looked on almost as a specific. "Sarcoma of the retina" is called a very rare and interesting condition; but under its old name of "Glioma," which the cases quoted at once suggest, it has not the same significance. The X-rays receive a large share of attention, and several cases of location of foreign bodies by their aid are carefully reported, and the methods given, but these are so complicated and the mathematics so severe as almost to stagger the reader. There is a fair report of a case of "toxic amblyopia" with many sections of the optic nerves, which seem at least to make the site of the lesion quite clear. Altogether this is a good book.

**Transactions of the American Laryngological Association.** New York: D. Appleton and Company. 1898.—This Association got through a great deal of good work in its three-day session, and much benefit may be obtained by reading this volume of Transactions. Dr. Charles H. Knight, in his presidential address, justly claimed that the Association is not carried away by fads of the moment, while at the same time it is ready to give judicial consideration to all new theories or modes of treatment.

**Shaw's Manual of the Vaccination Law.** By a Barrister-at-Law. Sixth Edition. Pp. xii., 148. London: Shaw & Sons. 1898.—The Vaccination Act of 1898 has necessitated a new edition of this excellent Manual, which contains all the statute law which will be in force on the subject at the beginning of 1899. The sections have appended to them a commentary which includes all the decisions of the courts bearing on them, and a very full index enables the facts to be very easily reached. A distinctive feature of this edition is a valuable introduction giving an historical statement of the law, with an epitome of the principal conclusions of the Royal Commission on which the new enactments are based. The 1898 Act will greatly increase the work and responsibilities of Public Vaccinators, who will not in a majority of cases find the "minimum" fees authorised by the Local Government Board adequate remuneration. Time alone can demonstrate the ultimate effect of the "tremendous experiment" of the recent legislation. One of two things must happen. The "conscientious objector" will prove more amenable to rational arguments now that his "grievance" is taken from him, or a severe epidemic of small-pox will cause the logic of facts to be brought fully home to him.

**Handbook on the Workmen's Compensation Act, 1897.** By M. Roberts-Jones. Fifth Edition. Pp. 82. Cardiff: Western Mail, Limited. 1898.—Whatever may be one's opinion of the justice of this Act, and of its provisions for the compensation for accidents, this must not be taken into consideration in noticing Mr. Roberts-Jones's book. That before the Act came into operation a fifth edition was called for, is evidence that the explanations and references given by the author were appreciated, probably both by the masters and the men. The general opinion, however, is that no one can foretell how the Act will work. Employers certainly do not look on it with favour, nor do we think they will get much relief to their anxiety from Mr. Roberts-Jones's book. The work is an excellent one, and should be in the hands of all employers and employed, as well as the Medical Referees appointed under the Act.

**Burdett's Hospitals and Charities.** London: The Scientific Press Limited. 1898.—Sir Henry Burdett, as perhaps might have been expected, has launched forth in his annual in praise of the Prince of Wales's Fund, and plays havoc with the persons who cry out that the fund stayed the ordinary flow of charity to the smaller institutions. We do not always accept Sir Henry's statements, or, perhaps we should say, opinions. We admit the easy flow of his rhetoric and the convincing manner of his argument, but for all that we remain unconvinced of his facts. Perhaps we have hardened our hearts too much. If an unfortunate individual happens to disagree on such a subject as the realisation of some reform of hospital abuse, he is pulverised with lengthy arguments and deluged, if not drowned, with figures. The useful part of the annual, *i.e.* the statistical part, is as good as ever.

**Year-Book of the Scientific and Learned Societies of Great Britain and Ireland.** London: Charles Griffin and Company, Limited. 1898.—We have with pleasure noticed this book in former years, and have advocated a more comprehensive index; we still think that if the names of the authors and titles of papers could be included in the index, the work would be more useful, and would be consulted by a larger number of persons as a work of reference. If a proof was sent to each secretary or other officer of the societies mentioned in the work, it would obviate some of the minor inaccuracies we notice on looking through it.

**Catalogue of Lewis's Medical and Scientific Library.** New Edition. London: Lewis's Library. 1898.—The title of this book speaks for itself. It is conveniently and usefully arranged with separate lists of authors and subjects, and forms an easy work of reference. It is revised to the end of 1897, and in its present form should be useful to the present-day reader, either in purchasing for his own library, or as a subscriber to Lewis's Library which offers him many advantages.

**Sixty-third Annual Report of the British Medical Benevolent Fund, for the Year 1897.** London: Morton & Burt.—We learn that, "in spite of every possible effort made by the committee and its officers to increase the receipts in this [the grant] department, they always fall very far short of meeting the great and increasing number of distressing appeals for help which are each month brought before the committee. . . . There are now at the close of the year an unusually large number of very urgent cases waiting for relief." The subscriptions for 1897 show a falling off of over £200 as compared with the previous year; on the other hand, there was a small increase in the donations. "What the fund most urgently requires is an increase in its regular annual subscribers." This charity appeals so especially to medical practitioners as to almost constitute a first claim on their benevolence, and if they fully knew the number and distressing character of the appeals for help, it would receive a larger measure of support from them. The hon. local secretary for this district is Dr. J. Michell Clarke, who would be glad to increase the number of local annual subscribers. If every medical man gave even the small subscription of five shillings, that would mean a considerable gain to the funds of the Society.

**The Medical and Surgical "Review of Reviews."** London: The Medical and Surgical "Review of Reviews," Ltd.—This monthly periodical, which began its existence in October, is not intended to rival or in any way take the place of the established ones, but to be an index and guide to those already in existence—a focus of the theoretical and practical advancement of medicine throughout the world. Every book of importance will be reviewed, and an annual index will be provided. We wish the editor, whose name was previously unknown to us, all success in his effort.

**Janus.** London: Williams and Norgate.—This magazine, now beginning its third year, presents many novel and attractive features. It is an international bi-monthly devoted to the history of medicine and to the study of diseases, with particular reference to their geographical distribution. Especial attention is given to military and naval medicine, and to the reviewing of medical books and magazines of all countries. With a large staff of correspondents in almost every known country, amongst whom are many well-known names, the success of the venture should be assured. The articles are written chiefly in English, French, German, and Italian. The Journal is published in Amsterdam, under the editorship of Dr. H. F. A. Peypers. A recent number contains some very fine prints illustrating a paper on Beri-Beri. Dr. J. F. Payne contributes some letters and fragments of Thomas Sydenham, some of which have not previously been published. Plague is dealt with by Drs. Matignon and Höfler, and African diseases by Dr. T. Brault, of Algiers. Other interesting articles are "The Early Days of Anæsthesia," by Dr. Cabanès, and some "Experiments with Dr. Unna's New Method of Treating Leprosy," by Dr. J. A. Voorthuis.

**Illustrirte Rundschau der medicinisch-chirurgischen Technik.** Berne: K. J. Wyss.—This international quarterly journal, the first number of which appeared in February, 1898, is a useful account of recent inventions and improvements in clinical methods, operations, and treatment other than that by drugs. New instruments are briefly described and figured in numerous woodcuts. There are separate sections devoted to internal medicine, to general surgery, to surgery of the trunk, limbs, and pelvis, and to surgery of the throat and sense organs. In each of these is a list of the recent papers on treatment and methods of diagnosis of the various diseases, with abstracts of the more important ones. Thus we find under internal medicine, not only references to the most recent articles of a practical character, but a summary of new work on the treatment of ataxy by mechanical means, on the examination of urine for tubercle bacilli, and on the use of currents of high frequency. Orthopædic and obstetric methods occupy considerable space. The editor, Dr. Gustav Beck, has indeed marked out for himself a new field, and the magazine offers to the busy practitioner a ready reference to the best of the suggestions and methods which are scattered over the medical journals of the world. The printing and illustrations are clear and attractive.

**Public Health.** London: The Rebman Publishing Company, Limited.—Our exchange-list has received the addition of this, which is the Journal of the Incorporated Society of Medical Officers of Health. A new volume was begun in October. We may mention that in the numbers before us Dr. J. N. Cook's paper on "The Causes of Failures of English Preventive Measures in India" in regard to the Plague, and Dr. A. K. Chalmers's careful "Inquiry into the Vital Statistics of School Ages," are of special interest. As the discussions which follow the papers read at branch meetings are often of suggestive value, we should like to see them reported. We do not admire the arrangement of the list of contents, which should be printed entire on the cover.

**Archiv für Verdauungs-Krankheiten.** Berlin: S. Karger.—This periodical, which is now in its fourth volume, deals with disorders or diseases of digestion, nutrition, and with dietetics, and has in addition a list of the current literature of the subject, with critical abstracts of the important papers. The original compilations in the first number of the present volume comprise a short note on the diagnostic value of the enumeration of the red blood-cells by Prof. F. P. Henry, of New York; observations on the occurrence of alimentary glycosuria in diseases of the liver, by Dr. Bierens de Haan; a paper on the histology of the stomach-glands in conditions of hyperacidity of the gastric juice. An article by Dr. Peltyn deals with digestion of proteids under the influence of solutions of the haloid salts. Dr. J. Boas, who edits the work, has a paper on stenosis of the pylorus due to simple hypertrophy and its treatment, and Dr. Westphalen describes a case of adhesions between the liver and colon. All these papers are good and deserving of careful study. The current literature is very completely dealt with; the abstracts are well done and sufficiently full. Workers at this branch of medicine will find this journal of very great value, if not indispensable, to them. We are very glad to have it on our exchange list.

**Letts's Medical Diary for the Year 1899.** London: Cassell & Company, Limited.—Two forms of this well-known diary have reached us. The less expensive is bound in cloth, and affords space for fifty-four patients; the other, an attractive book in French morocco in pocket-book fashion, has accommodation for double the number, but, having a thinner paper, is practically the same thickness as its companion. Both books have a large amount of information useful to doctors.

**Wright's Improved Physicians', Surgeons', and Consultants' Visiting List, 1899.** Bristol: John Wright & Co.—We have received two varieties of this list, one of which has the ordinary dated arrangement; the other has the "perpetual" form. The dates are printed at the bottom as well as at the top of the pages, and alternate lines are ruled in red to aid the eye in rapid reference. These elegant pocket-books are of convenient size, and should prove very popular.

**Wellcome's Medical Diary and Visiting List. 1899.** London: Burroughs, Wellcome & Co.—Less than half of this handsome volume of over 400 pages is occupied with the visiting-list. The other portion contains therapeutic notes referring to the drugs which are included in the repertory of this enterprising firm. There is also a variety of other useful information, which the practitioner will find it convenient to have within easy reach.

**Ephemeris Pharmacologica, 1899.** London: Oppenheimer, Son & Co., Ltd.—In addition to a considerable amount of information concerning drugs and other things of medical interest, this volume has a visiting-list arranged on the undated "perpetual" system. The work is issued in very neat and handy form.

